# Identification of differentially expressed genes in SHSY5Y cells exposed to okadaic acid by suppression subtractive hybridization

**DOI:** 10.1186/1471-2164-13-46

**Published:** 2012-01-27

**Authors:** Vanessa Valdiglesias, Juan Fernández-Tajes, Eduardo Pásaro, Josefina Méndez, Blanca Laffon

**Affiliations:** 1 Toxicology Unit, Psychobiology Department, University of A Coruña, Edificio de Servicios Centrales de Investigación, Campus Elviña s/n, 15071 A Coruña, Spain; 2 Department of Cell and Molecular Biology, University of A Coruña, Faculty of Sciences, Campus A Zapateira s/n, 15071 A Coruña, Spain

## Abstract

**Background:**

Okadaic acid (OA), a toxin produced by several dinoflagellate species is responsible for frequent food poisonings associated to shellfish consumption. Although several studies have documented the OA effects on different processes such as cell transformation, apoptosis, DNA repair or embryogenesis, the molecular mechanistic basis for these and other effects is not completely understood and the number of controversial data on OA is increasing in the literature.

**Results:**

In this study, we used suppression subtractive hybridization in SHSY5Y cells to identify genes that are differentially expressed after OA exposure for different times (3, 24 and 48 h). A total of 247 subtracted clones which shared high homology with known genes were isolated. Among these, 5 specific genes associated with cytoskeleton and neurotransmission processes (NEFM, TUBB, SEPT7, SYT4 and NPY) were selected to confirm their expression levels by real-time PCR. Significant down-regulation of these genes was obtained at the short term (3 and 24 h OA exposure), excepting for NEFM, but their expression was similar to the controls at 48 h.

**Conclusions:**

From all the obtained genes, 114 genes were up-regulated and 133 were down-regulated. Based on the NCBI GenBank and Gene Ontology databases, most of these genes are involved in relevant cell functions such as metabolism, transport, translation, signal transduction and cell cycle. After quantitative PCR analysis, the observed underexpression of the selected genes could underlie the previously reported OA-induced cytoskeleton disruption, neurotransmission alterations and *in vivo *neurotoxic effects. The basal expression levels obtained at 48 h suggested that surviving cells were able to recover from OA-caused gene expression alterations.

## Background

Okadaic acid (OA) is a marine toxin produced by several dinoflagellate species. It was firstly isolated from the black sponge *Halichondria okadai *[1] and is frequently found in several types of molluscs usual in the human diet as those from *Mytilus *or *Ostrea *genus. The ingestion of OA-contaminated shellfish results in a syndrome known as diarrhoeic shellfish poisoning (DSP) which is characterized by severe gastrointestinal symptoms including nauseas, vomit, diarrhoea and abdominal ache [2]. Although fatalities associated with DSP-contaminated shellfish have not been reported, this intoxication has become a serious problem for public health and for the economy of aquaculture industries in several parts of the world [3].

OA was found to be a very potent tumour promoter in two-stage carcinogenesis experiments *in vivo *involving mouse skin [4] or mucosa of the rat glandular stomach [5]. OA was also reported to induce different genotoxic, cytotoxic, and embryotoxic effects such as micronuclei [6-8], oxidative DNA damage [9,10], DNA strand breaks and alterations in DNA repair [11], mitotic spindle alterations [12,13], apoptosis [14,15], cell cycle disruptions [15,16], anomalies of the embryonic development [17] and teratogenicity [18]. Besides, despite the fact that DSP toxins are not classified as neurotoxins [19], some previous studies have already reported neurotoxic effects induced by OA including neuronal apoptosis and cytoskeleton alterations [20,21], deficits in spatial memory [22] and also cognitive deficits in rodents [23].

On the basis on these and other previous studies, OA represents other potential threats to human health besides DSP, even at concentrations within the nanomolar range. It is well-known that OA can inhibit specifically the serine/threonine protein phosphatases 1 (PP1) and 2A (PP2A) [24]; the number of physiological processes in which those phosphatases are involved is immense, including regulation of glycogen metabolism and coordination of the cell cycle and gene expression [25]. So this role of phosphatase inhibition by OA could explain most of the cell effects induced by this toxin [26]. However the number of controversial data in the literature continues increasing and further investigations on biochemical and molecular OA action mechanisms are required since the fact that non-phosphatase targets for OA are not known does not mean that they do not exist [3]. In fact, the existence of OA binding proteins other than phosphatases was demonstrated in several marine organisms [27,28].

In this study, a suppression subtractive hybridization (SSH) approach was used to identify genes differentially expressed in SHSY5Y cells in response to OA exposure at different times (3, 24 and 48 h). Sequences obtained by SSH were used to search for homology/identity to nucleotide and protein databases. Furthermore, differential expression patterns of 5 selected genes were also studied in OA-treated SHSY5Y cells at 3, 24 and 48 h by real-time PCR.

## Methods

### Cell culture and OA treatment

SHSY5Y cells (human neuroblastoma cell line) were obtained from the European Collection of Cell Cultures and cultured in nutrient mixture EMEM/F12 (1:1) medium with 1% non-essential aminoacids, 1% antibiotic and antimycotic solution and supplemented with 10% heat-inactivated foetal bovine serum, all from Invitrogen (Barcelona, Spain). The cells were incubated in a humidified atmosphere with 5% CO_2 _at 37°C. OA (CAS No. 78111-17-8) was purchased from Sigma-Aldrich Co. (Madrid, Spain) and dissolved in DMSO prior use.

Flasks with approximately 90% of confluence and 4 × 10^6 ^cells were chosen for the experiments. For the treatments, cells were incubated at 37°C for 3, 24 or 48 h in the presence of OA (100 nM) or the control dimethylsulfoxide (DMSO) at 1% of final volume.

### Total RNA isolation and cDNA synthesis for SSH

After OA treatments, total RNA was isolated from SHSY5Y cells with TRIZOL^® ^reagent (Invitrogen, Madrid, Spain) following the manufacturer's instructions, and then dissolved in nuclease-free water. RNA was quantified and quality checked using the NANODROP™ 1000 spectrophotometer (Thermo Scientific, Madrid, Spain). One microgram of total RNA from each sample was used as template to synthesize the first strand cDNA with the SMART™ PCR cDNA Synthesis Kit (Clontech, Madrid, Spain), a method for producing high quality cDNA from a low amount of starting material [29]. The double stranded cDNA was amplified with the same Kit according to the manufacturer's protocol.

### Construction of subtracted cDNA libraries

SSH was carried out with the PCR-Select™ cDNA subtraction kit (Clontech, Madrid, Spain), as described by the manufacturer. Briefly, the double stranded cDNAs obtained from the step described above were digested with the restriction enzyme *Rsa*I to obtain blunt-ends that are necessary for adaptor ligation. cDNA subtraction was carried out in two directions for the different exposure times. The forward subtracted libraries were made with the control cells cultured for 3, 24 or 48 h as the driver and OA-exposed cells (also cultured for 3, 24 or 48 h) as the tester. These forward reaction libraries were designed to produce clones that are overexpressed or up-regulated in OA-treated cells. The reverse libraries were made in the same way, but in this case the adapter ligated cDNA from OA-exposed cells were the driver and control cells were the tester. The reverse reaction library was designed to produce clones underexpressed or down-regulated in OA-treated cells. In either case the driver cDNA was added in excess during each hybridization to remove common cDNAs by hybrid selection and leaving overexpressed and novel tester cDNA to be recovered and cloned. The subtracted cDNA fragments were then inserted into yT&A^® ^cloning vector, transformed into *Escherichia coli *ECOS707 (strain JM109) competent cells, and plated on LB agar plates containing 100 μg/ml ampicillin, 100 μl IPTG (100 mM) and 20 μl X-gal (50 mg/ml). The yT&A^® ^cloning vector and the *E. coli *ECOS707 competent cells were purchased from Yeastern Biotech. Co., Ltd., (Taipei, Taiwan). From the six libraries, a total of 384 white recombinant colonies (corresponding to four 96-well plates) were picked.

### Sequencing of the subtracted cDNA clones and bioinformatics analysis

Sequencing of all the cDNA clones from the six SSH libraries was carried out using the BigDye^® ^Terminator v3.1 (Applied Biosystems) and an AB3730 sequencer (Applied Biosystems) at Secugen (Madrid, Spain). After excluding redundant and false-positive sequences, nucleic acid homology searches were performed against nucleotide databases at the National Center for Biotechnology Information (NCBI) using the Basic Local Alignment Search Tool (BLASTX and BLASTN) http://www.ncbi.nlm.nih.gov/BLAST to provide gene annotation. Homologies that showed identities over 60% and E-values of less than 1 × E^-10 ^with more than 100 nucleotides were considered to be significant. The differentially expressed genes identified through expression analysis were classified according to the definition of Gene Ontology (GO) http://www.geneontology.org/ related to the aspects of biological and molecular function.

### Differential screening of the subtracted libraries

With the aim of checking the level of background corresponding to common mRNAs in reverse and forward libraries we carried out a differential screening of subtracted libraries using the PCR select differentially screening kit (Clontech, Madrid, Spain), following the manufacturer's instructions. Briefly, PCR products from positive colonies were immobilized in nylon membranes and hybridized with forward- and reverse- probes. Those clones representing mRNAs truly differentially expressed should hybridize only with its corresponding forward-probe. Prior to hybridization forward- and reverse-probes were digested for removing adaptors. More than 90% of the clones tested resulted positive for the virtual Northern analysis (data not shown).

### Simple gene set enrichment analysis

A simple gene set enrichment analysis was performed using FatiGO tool (Babelomics 4.2 suite, http://babelomics.bioinfo.cipf.es/). FatiGO takes two lists of genes and convert them into two lists of GO annotations. Then a Fisher's exact test for 2 × 2 contingency tables is used to check for significant over-representation of GO annotations in one of the sets with respect to the other one. Multiple test correction is applied as a measure of control for false positives. In our case, we conducted two single gene set enrichment analysis for KEGG (Kyoto Encyclopedia of Genes and Genomes) pathways comparing our set genes from forward and reverse libraries with the rest of annotations in human genome (additional files 1 and 2).

### Quantitative PCR

Five EST identified from SSH were chosen for their specific analysis with real-time PCR. First strand synthesis was performed on 100 ng of the same total RNA samples prepared for SSH from OA-treated and control SHSY5Y cells (for 3, 24 and 48 h) using the Transcriptor First Strand cDNA Synthesis Kit (Roche). Oligonucleotide primers were designed based on the EST sequences determined for candidate differentially expressed genes using the web tool Universal ProbeLibrary (Roche) (Table 1). Quantitative PCR was run in triplicate using LightCycler^® ^SYBR green I Master Kit (Roche) and LightCycler^® ^480 Real-time PCR Detection System (Roche). The PCR conditions were 95°C for 10 s, 60°C for 10 s, and 72°C for 5 s, for 45 cycles, and final extension of 5 min. A subsequent melting temperature curve of the amplicon was performed. Efficiency of target amplification was optimised prior to running samples for each of the five primer pairs by assaying four primer concentrations (200, 150, 100 and 50 nM). The number of amplification steps required to reach the threshold cycle number (Ct) was computed using LightCycler software 1.5.0 (Roche). Constant Ct values were observed at a 100 nM final primer concentration for each of the primer pairs. Ct values were calculated from the standard curve, entered into the qBasePlus software [30] and used to generate an input file for genNorm software v3.5 [31]. GenNorm determined the most stable reference genes out of the panel of candidate genes using expression stability analysis by pair wise correlations. Following the results of the genNorm, TPR, ACTB and NM23A genes were selected and run separately in all experiments under the same conditions. Normalised cDNA levels of each gene were calculated using qBasePlus [30] once the most stable reference genes were determined. The expression levels of each gene of the 3 h libraries were normalised against both TPR and ACTB, 24 h libraries genes were normalized against both ACTB and NM23A, and 48 h libraries genes were normalized against both TPR and NM23A.

**Table 1:** Primers used in real-time PCR analysis

Genes	Primers (5'-3')	Product (bp)
NEFM	TGCCGGCTACATAGAGAAGG	62
	TCTCCGCCTCAATCTCCTTA	
TUBB	CCCTCTGTGTAGTGGCCTTT	68
	CCAGACAACTTTGTATTTGGTCA	
SEPT7	CACAATGTTCACCATTTTTCAAC	82
	TCATTGAAGTTAATGGCAAAAGG	
SYT4	AAAGTTGTAAGGGGCCAATTC	70
	ACCTCAGCTGCTACCACCAT	
NPY	CGCTGCGACACTACATCAAC	62
	CTCTGGGCTGGATCGTTTT	
TPR	CCACCGAGCGAGGTGATA	67
	AGAAGAAAGGCGAAGACCAGT	
ACTB	AAGTCCCTTGCCATCCTAAAA	91
	ATGCTATCACCTCCCCTGTG	
NM23A	ACATCCATTTCCCCTCCTTC	92
	AGCTTCCCTCCAAACTATGATG	

### Statistical analysis

Experimental data were expressed as mean ± standard error. Statistical analyses between groups were carried out using Student's *t*-test and a *P*-value of < 0.05 was considered significant. Statistical analysis was performed using the SPSS for Windows statistical package (version 16.0).

## Results and Discussion

The human SHSY5Y neuroblastoma cell line has been extensively used as a neuron model in many neurobiological, neurochemical, and neurotoxicological studies [32-37]. In the present study, we investigated the effects of OA, the main DSP toxin, on gene expression of SHSY5Y cells after 3, 24 and 48 h treatments.

### Identification of genes with different transcript levels in OA-exposed SHSY5Y cells

For each exposure time 2 subtracted cDNA libraries (one forward and one reverse) were obtained. We isolated a total of 114 subtracted clones from the forward libraries (Table 2) and 133 from the reverse libraries (Table 3).

**Table 2:** cDNAs selected from all 3 forward SHH libraries.

Gene name	Gene Symbol	E-value	**Genebank No**.	Number of genes
**3 h**				**24**

ATP synthase F0 subunit 6	ATP6	2.00E-173	XM_002345305	1
28S ribosomal RNA		1.00E-36	NR_003287	3*
B-cell receptor-associated protein 31	BCAP31	0.00E+00	NM_005745	1
calmodulin 2	CALM2	0.00E+00	NM_001743	1
chromosome 6 open reading frame 125	SGA-81M	0.00E+00	NM_032340	1
coiled-coil domain containing 56	CCDC56	0.00E+00	NM_001040431	1
ferritin	FTH1	2.00E-30	NM_002032	2*
FSHD region gene 1 family, member B	FRG1B	0.00E+00	NR_003579	1
H2A histone family, member Z	H2AFZ	8.00E-153	NM_002106	1
H3 histone, family 3A	H3F3A	1.00E-163	NM_002107	1
lysosomal protein transmembrane 4 alpha	LAPTM4A	0.00E+00	NM_014713	1
mitochondrial ribosomal protein L42	MRPL42	0.00E+00	NM_172178	1
fascin	FSCN1	3.00E-29	AAH07539	1
nerve growth factor receptor	TNFRSF16	6.00E-142	NM_206915	1
phosphoserine phosphatase	PSPH	2.00E-139	NM_004577	1
regulator of G-protein signaling 5	RGS5	0.00E+00	NM_003617	1
small nuclear ribonucleoprotein D3	SNRPD3	0.00E+00	NM_004175	1
superoxide dismutase 1	SOD1	0.00E+00	NM_000454	1
SWI/SNF related, matrix associated, actin dependent regulator of chromatin	SMARCE1	0.00E+00	NM_003079	1
ubiquitin C	UBC	5.00E-39	NM_021009	1
Unknown genes				1

**24 h**				**47**

calcyclin binding protein	CACYBP	0.00E+00	NM_014412	1
28S ribosomal RNA		1.00E-36	NR_003287	2*
calmodulin 2	CALM2	0.00E+00	NM_001743	1
casein kinase 1	CSNK1A1	0.00E+00	NM_001892	1
chromosome 14 open reading frame 147	SSSPTA	9.00E-93	NM_138288	1
claudin domain containing 1	CLDND1	0.00E+00	NM_019895	1
early growth response 1	EGR1	0.00E+00	NM_001964	1
ferritin	FTL	6.00E-161	NM_000146	1
glutamate-ammonia ligase (glutamine synthetase)	GLUL	0.00E+00	NM_002065	3*
glyceraldehyde-3-phosphate dehydrogenase	GAPDH	0.00E+00	NM_002046	1
GTP binding protein overexpressed in skeletal muscle	GEM	0.00E+00	NM_181702	2*
heat shock 70 kDa protein 8	HSPA8	0.00E+00	NM_006597	1
heat shock protein 90 kDa alpha (cytosolic)	HSP90AA1	0.00E+00	NM_005348	1
integrator complex subunit 6	INTS6	0.00E+00	NM_001039937	1
mab-21-like 1	MAB21L1	0.00E+00	NM_005584	1
methionine adenosyltransferase II, beta	MAT2B	9.00E-153	NM_182796	1
mitochondrial ribosomal protein L42	MRPL42	0.00E+00	NM_014050	1
NADH dehydrogenase (ubiquinone) 1	NDUFAB1	0.00E+00	NM_005003	1
neurofilament, medium polypeptide	NEFM	0.00E+00	NM_005382	1
nudix-type motif 5	NUDT5	0.00E+00	NM_014142	1
pituitary tumor-transforming 1	PTTG1	0.00E+00	NM_004219	1
polo-like kinase 1	PLK1	5.00E-103	NM_005030	1
ribosomal protein L15	RPL15	0.00E+00	NM_002948	1
ribosomal protein L23a	RPL23A	0.00E+00	NM_000984	1
RNA binding motif protein 7	RBM7	3.00E-111	NM_016090	1
shisa homolog 2	SHISA2	0.00E+00	NM_001007538	1
cytochrome b	CYTB	0.00E+00	XR_078322	1
SNF2 histone linker PHD RING helicase	SHPRH	0.00E+00	NM_001042683	1
TAF9 RNA polymerase II, TATA box binding protein (TBP)-associated factor	TAF9	0.00E+00	NM_003187	1
Tax1 (human T-cell leukemia virus type I) binding protein 1	TAX1BP1	0.00E+00	NM_006024	1
triosephosphate isomerase 1	TPI1	0.00E+00	NM_000365	2*
Unknown genes				11

**48 h**				**43**

actin, beta	ACTB	0.00E+00	NM_001101	3
adenylate kinase domain containing 1	AKD1	0.00E+00	NM_145025	1
ADP-ribosylation factor-like 6 interacting protein 1	ARL6IP1	0.00E+00	NM_015161	1
CD58 molecule	CD58	0.00E+00	NR_026665	1
clusterin	CLU	0.00E+00	NM_203339	1
cyclin-dependent kinase inhibitor 1C	CDKN1C	0.00E+00	NM_000076	1
cytochrome c oxidase subunit III	MT-CO3	6.00E-76	XM_002342023	3*
family with sequence similarity 32	FAM32A	0.00E+00	NM_014077	1
ferritin	FTL	0.00E+00	NM_000146	1
glutamate-ammonia ligase	GLUL	0.00E+00	NM_002065	1
metallothionein 1X	MT1X	2.00E-128	NM_005952	1
metastasis associated lung adenocarcinoma transcript	MALAT1	0.00E+00	NR_002819	6*
microtubule-associated protein	MAPRE2	0.00E+00	NM_014268	1
M-phase phosphoprotein 8	MPHOSPH8	0.00E+00	NM_017520	1
nudix -type motif 5	NUDT5	0.00E+00	NM_014142	1
prostaglandin reductase 1	PTGR1	0.00E+00	NM_012212	1
serine incorporator 3	SERINC3	0.00E+00	NM_198941	1
cytochrome b	CYTB	0.00E+00	XR_078322	1
solute carrier family 25	SLC25A4	0.00E+00	NM_001151	1
S-phase kinase-associated protein 1	SKP1	0.00E+00	NM_170679	1
TIMP metallopeptidase inhibitor 3	TIMP3	3.00E-86	NM_000362	1
transcription elongation factor B (SIII)	TCEB1	3.00E-139	NM_005648	1
translocase of outer mitochondrial membrane 5	TOMM5	0.00E+00	NM_001001790	1
tubulin, beta 2C	TUBB2C	2.00E-120	NM_006088	1
tubulin, delta 1	TUBD1	2.00E-81	NM_016261	1
tumor necrosis factor, alpha-induced protein 6	TNFAIP6	0.00E+00	NM_007115	1
tyrosine 3-monooxygenase/tryptophan 5-monooxygenase activation protein	YWHAB	8.00E-110	NM_003404	1
Unknown genes				7

*Only one GenBank identity accession number and the highest Blastp E-value were given here because of the table capacity limitation.

**Table 3:** cDNAs selected from all 3 reverse SHH libraries.

Gene name	Gene Symbol	E-value	**Genebank No**.	Number of genes
**3 h**				**35**

ferritin	FTL	0.00E+00	NM_000146	1
phosphoglycerate kinase 1	PGK1	0.00E+00	NM_000291	1
SMT3 suppressor of mif two 3 homolog 2	SUMO2	5.00E-115	NM_001005849	1
ribosomal protein S14	RPS14	2.00E-52	NM_001025071	1
ATPase	ATP6V0B	1.00E-47	NM_001039457	1
tubulin, beta 2A	TUBB2A	2.00E-27	NM_001069	1
actin, beta	ACTB	0.00E+00	NM_001101	1
yippee-like 5	YPEL5	3.00E-47	NM_001127401	1
translation elongation factor	EEF1A1	0.00E+00	NM_001402	1
early growth response 1	EGR1	0.00E+00	NM_001964	1
glyceraldehyde-3-phosphate dehydrogenase	GAPDH	0.00E+00	NM_002046	1
proteasome 26S subunit, ATPase, 5	PSMC5	0.00E+00	NM_002805	1
signal sequence receptor, alpha	SSR1	1.00E-130	NM_003144	1
protein disulfide isomerase family A, member 6	PDIA6	0.00E+00	NM_005742	1
malate dehydrogenase	MDH1	0.00E+00	NM_005917	2*
ubiquinol-cytochrome c reductase	UQCRH	0.00E+00	NM_006004	1
translocase of inner mitochondrial membrane	TIMM17A	0.00E+00	NM_006335	1
heat shock 70 kDa protein 8	HSPA8	0.00E+00	NM_006597	2*
peroxiredoxin 3	PRDX3	0.00E+00	NM_006793	1
Sec61 beta subunit	SEC61B	0.00E+00	NM_006808	1
CD24 molecule	CD24	0.00E+00	NM_013230	1
striatin, calmodulin binding protein 3	STRN3	0.00E+00	NM_014574	1
nucleolar protein 11	NOL11	6.00E-158	NM_015462	1
cleavage and polyadenylation factor subunit	PCF11	0.00E+00	NM_015885	1
zinc finger, CCHC domain	ZCCHC17	3.00E-138	NM_016505	1
homolog 2, suppressor of mek1	SMEK2	0.00E+00	NM_020463	1
5-azacytidine induced 2	AZI2	2.00E-161	NM_022461	1
anaphase promoting complex subunit 13	ANAPC13	2.00E-94	NR_024401	1
cytochrome c oxidase subunit II	MT-CO2	4.00E-150	XR_078889	1
NADH dehydrogenase subunit 4	MT-ND4	7.00E-54	ADG46850	1
Unknown genes				3

**24 h**				**44**

cholinergic receptor, nicotinic, alpha 3	CHRNA3	0.00E+00	NM_000743	1
ribosomal protein L3	RPL3	0.00E+00	NM_000967	1
ribosomal protein S6	RPS6	0.00E+00	NM_001010	1
actin, beta	ACTB	0.00E+00	NM_001101	1
casein kinase 2	CSNK2B	4.00E-176	NM_001320	1
eukaryotic translation elongation factor 1 alpha 1	EEF1A1	0.00E+00	NM_001402	1
calmodulin 2	CALM2	0.00E+00	NM_001743	4*
septin 7	SEPT7	0.00E+00	NM_001788	1
LIM domain only 1 (rhombotin 1)	LMO1	0.00E+00	NM_002315	1
proteasome beta type, 6	PSMB6	0.00E+00	NM_002798	1
signal recognition particle 9 kDa	SRP9	0.00E+00	NM_003133	1
translocated promoter region	TPR	0.00E+00	NM_003292	1
regulator of G-protein signaling 5	RGS5	0.00E+00	NM_003617	1
NADH dehydrogenase Fe-S protein 2	NDUFS2	0.00E+00	NM_004550	1
myeloid/lymphoid or mixed-lineage leukemia	MLLT11	4.00E-143	NM_006818	1
stathmin-like 2	STMN2	0.00E+00	NM_007029	1
component of oligomeric golgi complex 4	COG4	0.00E+00	NM_015386	1
RNA-binding region containing 3	RNPC3	0.00E+00	NM_017619	1
zinc finger, matrin type 5	ZMAT5	0.00E+00	NM_019103	1
synaptotagmin IV	SYT4	0.00E+00	NM_020783	2*
non-metastatic cells 1, protein NM23A	NM23A	0.00E+00	NM_198175	1
cytochrome c oxidase subunit III	MT-CO3	0.00E+00	XM_002342023	4*
ATP synthase F0 subunit 6	MT-ATP6	0.00E+00	XM_002345305	4*
cytochrome c oxidase subunit II	MT-CO2	5.00E-149	XR_078889	7*
NADH dehydrogenase subunit 4	MT-ND4	0.00E+00	XR_078993	1
Unknown genes				2

**48 h**				**54**

glutathione S-transferase	GSTP1	0.00E+00	NM_000852	1
neuropeptide Y	NPY	0.00E+00	NM_000905	1
ribosomal protein L3	RPL3	8.00E-120	NM_000967	2*
ribosomal protein L4	RPL4	0.00E+00	NM_000968	1
ribosomal protein L27a	RPL27A	0.00E+00	NM_000990	1
ribosomal protein L31	RPL31	1.00E-175	NM_000993	1
ribosomal protein S4	RPS4X	0.00E+00	NM_001007	1
chromosome 5 open reading frame 13	C5orf13	3.00E-85	NM_001142478	1
adenylate kinase 2	AKA2	7.00E-127	NM_001625	1
calmodulin 2	CALM2	0.00E+00	NM_001743	4*
translation initiation factor 4E	EIF4E	0.00E+00	NM_001968	1
lactate dehydrogenase B	LDHB	0.00E+00	NM_002300	1
NADH dehydrogenase (ubiquinone) 1	NDUFC1	2.00E-100	NM_002494	1
signal recognition particle 9 kDa	SRP9	0.00E+00	NM_003133	2*
regulator of G-protein signaling 5	RGS5	2.00E-68	NM_003617	4*
DNAJC25-GNG10 readthrough transcript	DNAJC25-GNG10	2.00E-166	NM_004125	1
guanine nucleotide binding protein	G protein	0.00E+00	NM_004126	1
sperm associated antigen 7	SPAG7	0.00E+00	NM_004890	1
heat shock protein 90 kDa	HSP90AA1	2.00E-166	NM_005348	1
chaperonin containing TCP1, subunit 3	CCT3	0.00E+00	NM_005998	1
ATP synthase	ATP5L	9.00E-140	NM_006476	1
mortality factor 4 like 1	MORF4L1	5.00E-96	NM_006791	1
myeloid/lymphoid or mixed-lineage leukemia	MLLT11	0.00E+00	NM_006818	1
transmembrane emp24-like trafficking protein	TMED10	0.00E+00	NM_006827	1
dickkopf homolog 1	DKK1	4.00E-70	NM_012242	1
signal peptidase complex subunit 1	SPCS1	0.00E+00	NM_014041	1
mitochondrial ribosomal protein L42	MRPL42	5.00E-142	NM_014050	1
HIG1 hypoxia inducible domain family	HIGD1A	0.00E+00	NM_014056	1
signal peptidase complex subunit 2	SPCS2	0.00E+00	NM_014752	1
mitochondrial ribosomal protein S7	MRPS7	4.00E-156	NM_015971	1
splicing factor 3B	SF3B14	0.00E+00	NM_016047	1
hematological and neurological expressed 1	HN1	5.00E-168	NM_016185	1
transmembrane protein 9	TMEM9	0.00E+00	NM_016456	1
chromosome 20 open reading frame 3	APMAP	0.00E+00	NM_020531	1
synaptotagmin IV	SYT4	0.00E+00	NM_020783	1
ribosomal protein L41	RPL41	0.00E+00	NM_021104	1
transmembrane protein 167A	TMEM167A	0.00E+00	NM_174909	1
tubulin, beta	TUBB	6.00E-101	NM_178014	1
THAP domain containing 5	THAP5	6.00E-154	NM_182529	1
K(lysine) acetyltransferase 5	KAT5	0.00E+00	NM_182709	1
gonadotropin-releasing hormone	GNRHR2	0.00E+00	NR_002328	1
cytochrome c oxidase subunit II	MT-CO2	1.00E-151	XR_078889	1
Unknown genes				4

*Only one GenBank identity accession number and the highest Blastp E-value were given here because of the table capacity limitation.

**Figure 1:**
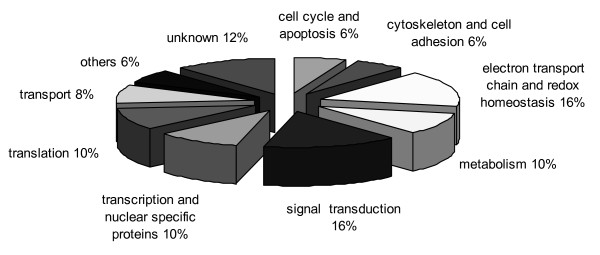
**Distribution by associated function of genes altered after 3 h OA treatment**.

These characterized genes were associated with various functions including metabolism, signal transduction, and cytoskeleton and cell adhesion. The genes altered after the 3 h OA treatment were related to electron transport chain and redox homeostasis, signal transduction, metabolism, transcription, translation, cell cycle and apoptosis, and cytoskeleton and cell adhesion (Figure 1). Most of these genes are apparently involved in metabolism including electron transport chain and redox homeostasis. A few studies have previously reported the OA effects on the cell metabolism. Cable *et al. *[38] observed that OA affected the heme metabolism of human hepatic cell lines. Also, Shisheva and Shechter [39] showed that OA mimicked some of insulin bioeffects stimulating the glucose and lipid metabolism in rat adipocytes, and Tanti *et al. *[40] found that glycolysis was stimulated and glucose transport was increased after OA treatment in mouse skeletal muscle. More recently, another study showed that OA depressed the metabolic rate of rat hepatocytes and changed glucose uptake in these cells [41]. Related to electron transport chain, OA was previously found to induce alterations in mitochondrial membrane potential [42] and increased oxidative stress in the rat brain after intracerebroventricular injection [43], and in different cell types *in vitro *[10,44]. The altered expression levels in genes related to cell metabolism and electron transport chain found in this study could help to explain the effects described in all these works. Besides, 8% of the genes altered after the 3 h OA treatment were related to cellular transport processes. OA was previously found to interfere in the secretion of newly synthesized proteins and exocytosis in rats [45]; both effects could be related to the expression alterations found in the present study.

**Figure 2:**
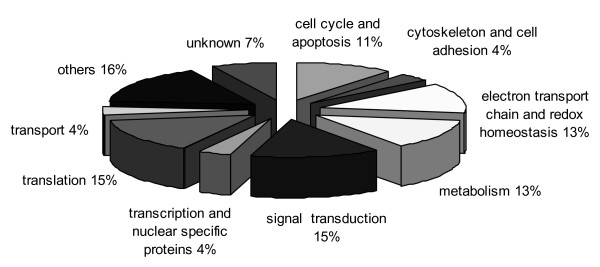
**Distribution by associated function of genes altered after 24 h OA treatment**.

When cells were treated with OA for 24 h, the obtained genes were also categorized into different groups including translation, signal transduction, electron transport chain and redox homeostasis, metabolism, cell cycle and apoptosis, transcription and nuclear specific proteins, transport, and cytoskeleton and cell adhesion (Figure 2). Similar to the 3 h OA treatment, an important number of these genes are involved in metabolism including electron transport chain, but also a great percentage of genes related to translation were observed. The expression alterations found in the genes involved in processes of translation and transcription might be related to the previously reported OA-induced inhibition of protein synthesis [45,46].

**Figure 3:**
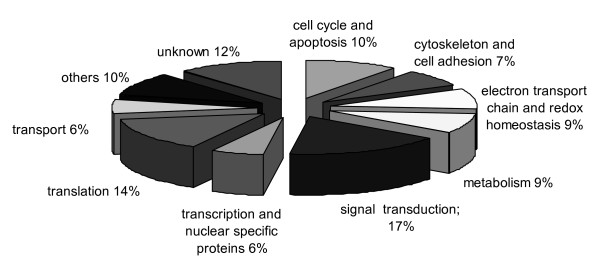
**Distribution by associated function of genes altered after 48 h OA treatment**.

Among the genes altered after the 48 h OA treatment, most were related to signal transduction, translation, cell cycle and apoptosis, electron transport chain and redox homeostasis, metabolism, cytoskeleton and cell adhesion, transcription and nuclear specific proteins, and transport (Figure 3). Fewer genes related to metabolism and transcription were found altered at 48 h, but similarly to 24 h an important percentage of altered genes are involved in cell cycle and apoptosis. In another previous study, the gene expression alterations in mouse BALB/c3T3 cells after different OA treatment times (from 4 h to 24 days, 7.8 ng/ml) were evaluated by microarray analysis, and a total of 177 differentially expressed genes were identified [47]. The authors focused this study on the 31 genes found to be functionally involved in cell growth and/or maintenance, and observed that numerous genes associated with cell proliferation and cell cycle progression were down-regulated after OA treatment. Several genes related to apoptotic processes, some of them involved in the mitochondrial pathway of apoptosis, were also found to be altered. On the basis of their results, they concluded that multiple molecular pathways could be involved in OA-induced proliferation inhibition and apoptosis in these cells [47].

Two simple gene set enrichment analysis were performed using FatiGO tool to find which cellular KEGG pathways could be affected by OA exposure in SHSY5Y cells. The results obtained for the forward libraries revealed a total of 3 KEGG pathways altered: oocyte meiosis (hsa04114, adjusted *p*-value = 0.003), Parkinson's disease (hsa05012, adjusted *p*-value = 0.035), and cell cycle (hsa04110, adjusted *p*-value = 0.003). The genes corresponding to reverse libraries were significantly associated with KEGG pathways related to: glycolysis (hsa00010, adjusted *p*-value = 0.044), oxidative phosphorylation (hsa00190, adjusted *p*-value = 0.012), *Vibrio cholerae *infection (hsa05110, adjusted *p*-value = 0.044), pathogenic *Escherichia coli *infection (hsa05130, adjusted *p*-value = 0.044), Alzheimer's disease (hsa05010, adjusted *p*-value = 0.022), and ribosome (hsa03010, adjusted *p*-value < 0.001). Since most effects of OA come from binding to PP1 and PP2A, a possible explanation for the altered pathways could be the protein phosphatases inhibition induced by this toxin. In fact, inhibition of PP2A by OA has been previously demonstrated to increase tau phosphorylation, a pathological hallmark of Alzheimer's disease, in SHSY5Y cells [48].

Since OA was previously reported to induce several neurotoxic effects in mammalian cells [20-23] but the underlying mechanisms are still unknown, five specific genes associated with important neuronal structures and functions such as cytoskeleton and neurotransmission, were selected to confirm their expression levels in SHSY5Y cells by real-time PCR. Results obtained from these analyses are shown in Table 4.

**Table 4:** Gene expression after real-time PCR.

	Control	OA (100 nM)
	**3 h treatment**	

**NEFM**	0.7 ± 0.1	0.1 ± 0.0*
**TUBB**	1.6 ± 0.4	1.0 ± 0.4
**SEPT7**	1.7 ± 0.4	1.0 ± 0.1*
**SYT4**	1.4 ± 0.0	0.3 ± 0.0**
**NPY**	0.6 ± 0.2	0.3 ± 0.0*

	**24 h treatment**	

**NEFM**	1.6 ± 0.0	5.6 ± 0.6*
**TUBB**	1.5 ± 0.1	0.4 ± 0.0**
**SEPT7**	1.2 ± 0.1	0.9 ± 0.2
**SYT4**	2.8 ± 0.1	0.7 ± 0.2*
**NPY**	2.3 ± 0.3	1.3 ± 0.1*

	**48 h treatment**	

**NEFM**	1.3 ± 0.1	0.8 ± 0.3
**TUBB**	1.3 ± 0.1	1.5 ± 0.7
**SEPT7**	0.6 ± 0.0	0.7 ± 0.0
**SYT4**	0.9 ± 0.1	0.9 ± 0.0
**NPY**	1.1 ± 0.1	1.2 ± 0.1

*P < 0.05, **P < 0.01, significant difference with regard to the expression of reference gen.

### NEFM, TUBB2A and SEPT7 expression: OA effects on neuronal cytoskeleton

The key role of cytoskeletal organization in several important neural processes such as neurite outgrowth [49], synaptogenesis [50], structural polarity and neuronal shape [51], axonal transport [52], and neurotransmitter release [53] has been characterized. Cell shape and structural polarity are lost in neurodegenerative diseases and neural aging [54,55].

OA was previously reported to induce several cytoskeleton alterations in different cell systems [56,57]. It has been hypothesized that these alterations could be due to different mechanisms that involve disruption of F-actin and ⁄or hyperphosphorylation and activation of kinases that stimulate tight junction disassembly, but the exact molecular mechanism has not been elucidated yet [3].

The cytoskeleton is made up of three kinds of protein filaments: actin filaments (also called microfilaments), intermediate filaments (IF) and microtubules, and other associated proteins. NEFM is the human gene which encodes for the neurofilament medium polypeptide. Neurofilaments (NEFs) are a neuron-specific type of intermediate filament proteins (10 nm in diameter) found at especially high concentrations along the axons, where they regulate axonal diameter. NEFs consist of three proteins according to their molecular weight: light neurofilament (NEFL), medium neurofilament (NEFM), and heavy neurofilament (NEFH) [58]. NEFM gene is often employed as a marker of neuronal differentiation [59]. NEF protein levels are correlative to neurite outgrowth, and its gene expression is dramatically altered in neurodegenerative diseases, including Parkinson's disease and Alzheimer's disease [60]. NEF protein levels have also been suggested as a potential biomarker in organophosphorous neurotoxicity [36]. Furthermore, neurite outgrowth can be promoted by nerve growth factor (NGF) via the regulation of NEF gene expression and NEF protein phosphorylation [61]. In the present study a statistically significant underexpression of this gene was found after 3 h OA treatment, but an overexpression was observed after 24 h, and no effects after 48 h, suggesting that OA deregulates NEFM expression at the short term (within at least the first 24 h), but then it stabilizes and return to control levels. It was previously described that tight coordination of the expression of neurofilament subunits is integral to the normal development and function of the nervous system. Imbalances in their expression are implicated in the induction of neurodegeneration in which formation of neurofilamentous aggregates is central to the pathology [62]. To our knowledge, no studies were reported before on the expression of this gene in human neuronal cells exposed to OA; nevertheless incubation of human fibroblasts [63] or rat brain tumour cells [64] with OA promoted the hyperphosphorylation of major intermediate filaments (IF) proteins, leading to the disassembly of IF networks, solubilisation of IF proteins, and disruption of desmosomes.

Microtubules are involved in many cellular functions, including mitosis, intracellular transport, determination of cell morphology, and differentiation [65]. In neurons, microtubules participate actively in the initial steps of neuronal polarization, the organization of intracellular compartments, the modelling of dendritic spines and the trafficking of cargo molecules to pre-, post- or extrasynaptic domains [66]. Tubulin, the subunit protein of microtubules, is a heterodimer, with both α- and β-tubulin isotypes, differing from each other in amino acid sequence [67]. In our study, the expression levels of TUBB2A, the gene encoding for the tubulin β chain 2A, were analyzed in OA-treated neuronal cells. Data obtained from the real-time PCR analysis showed that this gene is down-regulated in OA-treated SHSY5Y cells at 3 h and 24 h, significantly only in the second case, but at 48 h its expression levels were not different from the control. Microtubules were found to be altered after OA exposure in some previous studies mainly due to the hyperphosphorilation of tau, a microtubule-associated protein which promotes microtubule enlargement. Inhibition of PP2A activity by OA was suggested to produce the abnormal tau hyperphosphorylation *in vivo *after hippocampal injection in rats [68] and *in vitro *in metabolically competent brain rats [69], in mouse hippocampal HT22 cell line [70] and in human neuronal NT2N and SHSY5Y cells [48,71]. Besides, Yano *et al. *[56] found that OA induces reorganization of microtubules in human platelets via the phosphorylation of a microtubule-associated 90 kDa protein, and Benitez-King *et al. *[72] showed that OA produces cytoskeletal disorganization and microtubule disruption in N1E-115 neuroblastoma cells, as described in other neuronal cell culture models and in rat brain [20,73,74]. TUBB2A was characterized primarily as a neuronal β-tubulin isotype [75] and possess a high expression level in brain, peripheral nerves and muscles [76]. Tubulin isotype composition may be a determinant factor on microtubule functions. Therefore, changes in expression levels of tubulin subtypes would alter the microtubule dynamics. In this sense, Falconer *et al. *[77] demonstrated that TUBB2 is preferentially incorporated into stable microtubules during neuronal differentiation, and Hoffman and Cleveland [78] reported that the isotype TUBB2 is polymerized more efficiently than other isotypes. The higher expression observed in several types of tumours [79] and in cancer cells resistant to microtubule-binding drugs [80] could be related with the more stability of TUBB2A isotype. The underexpression of TUBB2A observed in this work might contribute to cytoskeletal disruption effects of OA in a similar way, since the major isotype in neuronal cells and the more would be incorporated in a lesser extent to microtubules of SHSY5Y OA-exposed cells.

Septins are an evolutionarily conserved family of cytoskeleton GTP-binding proteins [81]. They play putative roles in cytokinesis, cellular morphogenesis, polarity determination, vesicle trafficking and apoptosis [82-85]. Septins have been identified in all eukaryotic cells. Although yeast septins are better understood, the function of mammalian septins remains largely undefined [86]. SEPT7 is a member of the septin family which is abundantly expressed in the central nervous system [81], but its functional role has not been reported yet [87]. However, a previous study showed that SEPT7 is critical for spine morphogenesis and dendrite development during neuronal maturation [87] and other study confirmed that SEPT7 directly interacts with CENP-E via the C-terminal coiled-coil region. This SEPT7-CENP-E interaction is critical for a stable CENP-E localization to the kinetochore and for achieving chromosome alignment at the equator [88]. SEPT7 has been also related to oncogenesis. After investigating SEPT7 expression in a large number of human glioma tissue samples, Jia *et al. *[86] found that the expression of this gene was generally down-regulated in gliomas or even absent in some high-grade tumours. Furthermore, they showed that SEPT7 induces cell apoptosis through down-regulation of Bcl-2 and up-regulation of caspase-3, and increased cell apoptosis also contributes to the inhibitory effect of SEPT7 on glioma cell growth. Other studies showed that SEPT7 was much less expressed in brain tumours than in normal brain tissues [81,89] and that neuroblastoma patients with higher SEPT7 mRNA expression might have better prognosis [90]. In our study a significant decrease in the SEPT7 expression levels were found at 3 h, however no differences with regard to the control cells were observed after 24 or 48 h OA treatment.

Therefore, the results obtained from the genes related to cytoskeleton evaluated in this study suggest that the cytoskeleton disruption induced by OA described in previous works are due not only to the hyperphosphorylation of specific proteins caused by phosphatases inhibition, but also to short term alterations (mainly down-regulation) in the expression levels of relevant genes involved in the maintenance of the cell structure as the TUBB2A, NEFM or SEPT7 genes. The fact that no effects of OA were observed after 48 h treatment in any of these genes could be related to cells ability to recover and return to their normal expression levels. However, cell viability was enormously reduced after 48 h OA treatment (microscopic observations). Thus, the absence of gene expression alterations found at that time might also be due to the fact that those cells intensely altered by OA in their gene expression at 3 and 24 h treatments underwent apoptosis or necrosis, being absent at 48 h.

### SYT4 and NPY expression: OA effects on synaptic neurotransmission

Synaptic neurotransmission is one of the most highly regulated of all vesicle trafficking events. Although many of the molecular components of synaptic vesicles, the presynaptic cytosol, and presynaptic plasma membrane have been identified, the mechanisms by which these components regulate stimulus evoked vesicle fusion and recycling are not completely understood yet. In this study, expression levels of two genes related to the neuronal signal transduction (SYT4 and NPY) after OA exposure were investigated.

The synaptotagmins (SYTs) are a family of proteins characterized by a short luminal NH_2 _terminus, one transmembrane region, and tandem C2A and C2B domains [91]. These synaptic proteins are also important in depolarization-induced, Ca^2+^-dependent fusion of the synaptic vesicles and presynaptic membrane [92]. Currently, it is thought that SYTs participate in the regulation of various steps during membrane fusion, primarily at the plasma membrane [93]. There are at least 17 SYTs isoforms that have the potential to act as modulators of membrane fusion events. SYT4 is particularly interesting since it has been found to be potentially involved in a wide variety of activities in the brain [94]; it is an immediate early gene that is up-regulated following neuronal depolarization [95] and maps to a region of human chromosome 18 associated with schizophrenia and bipolar disease [96]. Data obtained from real-time PCR showed that the SYT4 expression is inhibited by OA at 3 and 24 h exposure, but it recovers normal levels at 48 h treatment. In a previous study, loss of SYT4 results in a reduction of synaptic vesicles and a distortion of the Golgi structure in cultured hippocampal neurons [94]. Golgi disruptions were also found in rat pancreatic cells after OA exposure [45]. Besides, SYT4 affects a number of vesicle recycling properties in peptidergic nerve terminals in the posterior pituitary [97]. Interestingly, SYT4 also appears to play a role in the maturation of secretory granules in neuroendocrine cells [93], suggesting that it may also function in the movement of vesicles [91].

Neuropeptide Y (NPY) is a 36-amino acid peptide produced by neurons throughout the brain and by other secretor cells of the body. NPY has been associated with a number of physiological processes in the brain, including the regulation of energy balance, memory and learning, and epilepsy [98]. Similarly to SYT4, NPY expression levels after OA exposure were found to be down-regulated at 3 and 24 h, but expression levels similar to control were observed at 48 h. A deregulation of the hypothalamic NPY system has been proposed to be related to several pathological and pathophysiological states including cancer cachexia [99], hyperinsulinemia and hypercorticism [100], obesity and metabolic syndrome [101], and anorexia [102].

So far, no studies on SYT4 or NYP expression after OA exposure were reported, but several previous studies described neurotransmission alterations after OA exposure, and the down-regulation of genes involved in the synaptic processes found in this study could help to explain them. OA was found to inhibit mobilization of synaptic vesicles and depress Ca^2+ ^release from sarcoplasmic reticulum in mouse neuromuscular junctions [103], to disrupt synaptic vesicle trafficking in goldfish bipolar cells [104], and to interfere with the formation of synaptic vesicle clusters in nerve terminals of frog neuromuscular junctions [105]. *In vivo*, OA significantly reduces electrically induced inhibitory non-adrenergic, non-cholinergic (NANC) neurotransmission responses in the rat gastric fundus, while leaving direct muscular effects of the inhibitory NANC neurotransmitters vasoactive intestinal peptide and nitric oxide unaffected, suggesting a neural site of action [106]. It was also reported that presynaptic clusters of synaptic vesicles at the frog neuromuscular junction can be disrupted by exposure to OA [105]. Furthermore, our data involving genes related to neurotransmission could also underlie the OA effects previously reported on the rodent nervous system *in vivo *such as hyperexcitation [107], spatial memory deficit and neurodegeneration [22] and cognitive deficits [48]. Similarly to the results obtained for the cytoskeleton genes expression, the expression levels of both SYT4 and NPY were highly depressed at 3 and 24 h OA exposure, but they went back to basal levels after 48 h, suggesting that surviving cells were able to recover from OA-induced gene expression alterations.

## Conclusions

To elucidate the molecular mechanisms involved in the OA-induced neurotoxic effects, SSH was used in SHSY5Y cells to identify genes with altered expression level at designated treatment times in the promotion stage, including an early time point (3 h), a middle time point (24 h) and a late time point (48 h). A total of 247 known genes were found to be altered. At 3 h OA treatment genes altered are mainly involved in metabolism, including electron transport chain and transcription processes. At 24 and 48 h OA treatments, the percentage of genes related to translation, cell cycle and apoptosis increased. The percentage of genes related to signal transduction, cytoskeleton and metabolism was in general constant at the 3 treatment times.

The data obtained from SHH were confirmed by real-time PCR for 5 specific genes associated with neuronal cytoskeleton and neurotransmission: NEFM, TUBB2A, SEPT7, SYN4, and NPY. The expression levels of the three genes involved in cytoskeleton processes (NEFM, TUBB2A and SEPT7) were found to be altered at 3 and 24 h OA treatments. These alterations could help to explain the previously reported cytoskeleton modifications induced by OA including cell rounding, loss of stabilization of focal adhesions, loss of barrier properties, and loss of cell polarity [56,57,108-110]. The down-regulation observed at the short term (3 and 24 h) of the two genes participating in synaptic neurotransmission (SYT4 and NPY), might be the basis of several reported OA-induced neurotoxic effects [22,48,107]. No expression alterations were observed for any of the five studied genes at 48 h OA exposure, so surviving cells recovered their normal gene expression levels. In order to test whether current results are dependent on OA dose, similar experiments testing different OA concentrations are currently being carried out. Further investigations on the expression patterns of other relevant genes is required in order to completely understand the different effects induced by OA in these and other cells.

## Authors' contributions

VV performed the cell culture, prepared SSH libraries, carried out the sequence analyses, did the molecular work and drafted the manuscript. JFT was involved in the conceptualization, design, and implementation of all experiments; helped with the SSH libraries and molecular work and revised the manuscript. EP helped with the experiment design and drafting and revision of the manuscript. JM, the first author's (VV) Ph.D. co-supervisor, was involved in the conceptualization, design, and revision of the manuscript. BL, the other first author's (VV) Ph.D. co-supervisor, was involved in the conceptualization, design and implementation of experiments, and took an active part in data interpretation and writing of this manuscript. All authors read and approved the final manuscript.

## Supplementary Material

Additional file 1 **Single gene set enrichment analysis (KEGG pathways) of genes from forward libraries**. Excel spreadsheet showing the results obtained from FatiGO analysis of the genes upregulated in SSH. Click here for file

Additional file 2 **Single gene set enrichment analysis (KEGG pathways) of genes from reverse libraries**. Excel spreadsheet showing the results obtained from FatiGO analysis of the genes downregulated in SSH. Click here for file
